# VPA improves ferroptosis in tubular epithelial cells after cisplatin-induced acute kidney injury

**DOI:** 10.3389/fphar.2023.1147772

**Published:** 2023-04-19

**Authors:** Yan Li, Ke Li, Weihao Zhao, Haodong Wang, Xiaodong Xue, Xianghui Chen, Wantao Li, Peihao Xu, Kexin Wang, Pengfei Liu, Xuefei Tian, Rongguo Fu

**Affiliations:** ^1^ Department of Nephrology, The Second Affiliated Hospital of Xi’an Jiaotong University, Xi’an, Shaanxi, China; ^2^ School of Computer Science, National University of Singapore, Singapore, Singapore; ^3^ School of Medicine, Royal College of Surgeons in Ireland, Dublin, Ireland; ^4^ National & Local Joint Engineering Research Center of Biodiagnosis and Biotherapy, The Second Affiliated Hospital of Xi’an Jiaotong University, Xi’an, Shaanxi, China; ^5^ Section of Nephrology, Department of Internal Medicine, Yale University School of Medicine, New Haven, CT, United States

**Keywords:** ferroptosis, HDAC inhibitor, cisplatin, acute kidney injury, VPA

## Abstract

**Background:** As a novel non-apoptotic cell death, ferroptosis has been reported to play a crucial role in acute kidney injury (AKI), especially cisplatin-induced AKI. Valproic acid (VPA), an inhibitor of histone deacetylase (HDAC) 1 and 2, is used as an antiepileptic drug. Consistent with our data, a few studies have demonstrated that VPA protects against kidney injury in several models, but the detailed mechanism remains unclear.

**Results:** In this study, we found that VPA prevents against cisplatin-induced renal injury *via* regulating glutathione peroxidase 4 (GPX4) and inhibiting ferroptosis. Our results mainly indicated that ferroptosis presented in tubular epithelial cells of AKI humans and cisplatin-induced AKI mice. VPA or ferrostatin-1 (ferroptosis inhibitor, Fer-1) reduced cisplatin-induced AKI functionally and pathologically, which was characterized by reduced serum creatinine, blood urea nitrogen, and tissue damage in mice. Meanwhile, VPA or Fer-1 treatment in both *in vivo* and *in vitro* models, decreased cell death, lipid peroxidation, and expression of acyl-CoA synthetase long-chain family member 4 (ACSL4), reversing downregulation of GPX4. In addition, our study *in vitro* indicated that GPX4 inhibition by siRNA significantly weakened the protective effect of VPA after cisplatin treatment.

**Conclusion:** Ferroptosis plays an essential role in cisplatin-induced AKI and inhibiting ferroptosis through VPA to protect against renal injury is a viable treatment in cisplatin-induced AKI.

## 1 Introduction

Acute kidney injury (AKI) represents a clinical syndrome that is characterized by a rapid decline in renal function over a short period of time and can be caused by multiple pathological factors, including a variety of drugs (Hoste et al., 2018). Cisplatin is an important chemotherapeutic agent widely used for cancers, and its obvious side effect is nephrotoxicity, especially renal tubular epithelial cell apoptosis and necrosis (Fang et al., 2021). Although cisplatin has been used in patients for more than 40 years, its side effect of nephrotoxicity can be limited to approximately 30% of patients (Gómez-Sierra et al., 2018). Cisplatin nephrotoxicity can present a variety of clinical manifestations such as AKI, hypomagnesemia, tubular acidosis, albuminuria and chronic renal failure, among which AKI is the most common and serious side effect. The mechanism of cisplatin-induced AKI has not been fully clarified and the clinical therapy targeting cisplatin nephrotoxicity cannot completely prevent the occurrence of AKI (Holditch et al., 2019). This illustrates the need for research on viable prevention and treatment options for cisplatin-induced AKI for patients.

Previous research observed that oxidative stress induced by mitochondrial dysfunction and reactive oxygen species (ROS) are major causes of cell death in cisplatin-induced AKI(Chen et al., 2019; Mapuskar et al., 2019; Deng et al., 2020). Ferroptosis is a new iron-dependent cell death featured by massive iron accumulation and lipid peroxidation, which is a variant of cell apoptosis and necrosis (Mishima et al., 2020). Unlike other forms of apoptotic death, mitochondrial damaged and ROS accumulation in ferroptosis is critical since its first reported in 2012 (Dixon et al., 2012; Chen et al., 2021). Then, it was reported that glutathione peroxidase 4 (GPX4) is the significant enzyme that suppresses ferroptosis (Yang et al., 2014). And, acyl-CoA synthetase long-chain family member 4 (ACSL4) was considered as a mediator in ferroptosis in HK-2 cell induced by cisplatin (He et al., 2022; Kuwata et al., 2022). Our study in mice, as reported before (Hu et al., 2020; Zhao et al., 2020), showed that the expression of GPX4 was decreased and the expression of ACSL4 was increased in AKI. The finding indicated ferroptosis is involved in the development of cisplatin-induced AKI. However, further studies are needed to explore the treatment of cisplatin-induced AKI by inhibiting ferroptosis.

Valproic acid (VPA) is a well-tolerated anticonvulsive drug, which has been extensively studied as an antineoplastic agent and is considered primarily a classⅠhistone deacetylase (HDAC) inhibitor (Singh et al., 2021). The family of HDAC contains 18 genes, which are divided into class I to IV. Class Ⅰ includes HDAC 1, 2, 3, and 8, and is closely related to kidney disease. Recent evidence shows that HDAC1 and HDAC2 are frequently overexpressed in the injured kidneys that it is possible to improve renal function with HDAC inhibitor (Ranganathan et al., 2016; Liu, 2021). The reno-protective effects of VPA have been demonstrated in ischemia-reperfusion injury (IRI) (Zheng et al., 2014) and lipopolysaccharide (LPS) induced injury (Shang et al., 2010), unilateral ureteral obstruction (UUO) mice (Kawaoka et al., 2017), diabetic nephropathy (Khan et al., 2015; Sun et al., 2016), podocyte injury (Inoue et al., 2019) in the kidney. Only a few studies focus on the protection of VPA in cisplatin-induced AKI (Ma et al., 2017; Jiang et al., 2020; Zhu et al., 2020). Therefore, the precise mechanisms by which VPA protection against cisplatin-induced AKI have not been determined.

As such, it is not completely cleared whether the renal protective effect of VPA in AKI is achieved through ferroptosis. This study aims to explore the relationship between the renal protective effect of VPA on cisplatin-induced AKI and ferroptosis, and to provide solid evidence for the clinical study of drugs for cisplatin-induced AKI.

## 2 Materials and methods

### 2.1 Reagents and antibodies

Cisplatin was purchased from Aladdin (China). Valproic acid and ferrostatin-1 were purchased from MedChemExpress (United States). The BUN (Blood urea nitrogen assay kit), serum creatinine (Creatinine assay kit), reduced glutathione (GSH) assay kit, malondialdehyde (MDA) assay kit, and cell malondialdehyde (MDA) assay kit were obtained from Nanjing Jiancheng Bioengineering Institute (China). Mouse HDAC1 and HDAC2 Elisa kits were purchased from Sino Best Biological Technology Shanghai (China). A one-step TUNEL apoptosis assay kit and reactive oxygen species (ROS) assay kit were purchased from Beyotime Biotechnology (China). The propidium iodide (PI) staining kit was purchased from Beijing Solarbio science and technology (China). Anti-HDAC2 rabbit monoclonal antibody (ab32117), anti-Histone H3 rabbit polyclonal antibody (ab4729), anti-ACSL4 rabbit monoclonal antibody (ab155282), and anti-GPX4 rabbit monoclonal antibody (ab125066) were purchased from Abcam (United Kingdom). Anti-HDAC1 mouse monoclonal antibody (66085-1-Ig) and anti-GAPDH rabbit monoclonal antibody (60004-1-Ig) were purchased from Proteintech (China). The reagents and antibodies used in this study were summarized in Supplementary Tables S1, S2.

### 2.2 Human samples

Human samples were obtained from a renal biopsy of 3 cases of AKI patients (Patients with clinical diagnosis of AKI and pathological diagnosis of acute tubular injury (ATI) or acute tubular necrosis (ATN)) and adjacent tissues of 3 cases of renal cancer patients in the Second Affiliated Hospital of Xi’an Jiaotong University. All procedures were approved by the Clinical Ethics Committee of the Second Affiliated Hospital Xi’an Jiaotong University (NO. 2021-839).

### 2.3 Animals and experimental protocol

Male C57BL/6 mice were obtained from the Animal Care and Use Committee of Xi’an Jiaotong University. The animal experiments were conducted in accordance with the guidelines and regulations set forth by the Animal Care and Use Committee of Xi’an Jiaotong University (NO. 2020-1292). The mice were housed in a specific pathogen-free environment under controlled conditions, including a 12-h light/dark cycle, 22°C ± 1°C temperature, 55% humidity, and provided with free access to food and water.

A total of 6 seven/eight-week-old C57BL/6 male mice weighing 18–20 g were randomly divided into two groups: Sham group and Cisplatin group. Cisplatin (20 mg/kg dissolved in saline) was injected once in mice. Mice were sacrificed at 72 h after cisplatin treatment, and the serum and kidney tissues of mice were collected.

A total of 25 seven/eight-week-old C57BL/6 male mice weighing 20.5–25.2 g were randomly divided into five groups: Sham group (n = 5), Cisplatin (20 mg/kg dissolved in saline) group (n = 5), Sham + VPA (200 mg/kg dissolved in 10% DMSO and 90% (20% SBE-β-CD in saline)) group (n = 5), Cisplatin + VPA group (n = 5) and Cisplatin + Fer-1 (1 mg/kg dissolved in 10% DMSO and 90% (20% SBE-β-CD in saline)) group (n = 5), were administered intraperitoneally. The mice were randomly captured and labeled with ear tags. They were randomly divided into five groups according to the order of ear tags. Cisplatin was injected once in mice, while VPA and Fer-1 were injected once daily for five consecutive days before cisplatin. Mice were sacrificed at 72 h after cisplatin treatment, and the serum and kidney tissues of mice were collected.

### 2.4 Cell culture and treatment

Human proximal tubular epithelial cells (HK-2 cells) were procured from the China Center for Type Culture Collection (CCTCC, Wuhan, China) and cultured in DMEM/F12 (HyClone, Logan, UT, United States) supplemented with 10% FBS (ZETA LIFE, United States). The HK-2 cells were maintained in a cell incubator at 37°C with a humidified atmosphere containing 95% air and 5% CO_2_. The cells were divided into five groups, namely, Control group, Control + VPA group, Cisplatin group, Cisplatin + VPA group, and Cisplatin + Fer-1 group. The Control + VPA group and Cisplatin + VPA group were pretreated with 1 mM VPA before inducing cisplatin (10 μM). The Cisplatin + Fer-1 group was pretreated with 1 μM Fer-1 before cisplatin induction. Further, the HK-2 cells were divided into six groups, including NC group, NC + Cisplatin group, NC + Cisplatin + VPA group, GPX4 siRNA group, GPX4 siRNA + Cisplatin group, and GPX4 siRNA + Cisplatin + VPA group. The three small interfering RNA targeting human GPX4 mRNA were synthesized by GenePharma (China), and their sequences were listed in Supplementary Table S3. The HK-2 cells were co-transfected with the siRNA using Lipofectamine 2000 (Invitrogen, Carlsbad, United States) as per the manufacturer’s instructions.

### 2.5 Measurement of BUN, serum creatinine, MDA and GSH

Mice serum was collected 72 h after cisplatin treatment for quantification of blood urea nitrogen (BUN) levels and serum creatinine levels using detection kits. Malondialdehyde (MDA) levels and reduced glutathione (GSH) levels in the renal tissues of mice were measured using the corresponding detection kits by the manufacturer’s instructions. In addition, lysate of HK-2 cells was collected for the detection of GSH and MDA levels.

### 2.6 Renal tissue histopathological, immunohistochemistry (IHC), immunofluorescence staining (IF), and TUNEL assessment

Fresh mice renal tissues were collected, fixed immediately with formalin, and then embedded in paraffin, followed cut into a thickness of 3 μm sections, which were used for hematoxylin-eosin (H&E) staining, immunohistochemistry, immunofluorescence, and TUNEL fluorescent staining. The degree of renal tubulointerstitial damage in mice was assessed based on tubular dilation, cast formation, and tubular interstitial fibrosis. 10 fields of each mouse kidney tissue were examined as H&E-stained sections. The semiquantitative analysis was scored as follows: 0 = no lesion; 1 = lesion of <10% of the areas; 2 = lesion of 10%–25% of the areas; 3 = lesion of 25%–50% of the areas; 4 = lesion of >50% of the areas (Tian et al., 2020). The immunohistochemistry photos were analyzed using Image J software to determine their intensities. The brown areas were identified as positive, and the staining intensity was measured by calculating the integral optical density (IOD). Similarly, the intensities of immunofluorescence photos were also measured using the integral optical density (IOD). The cell death rate was quantified as well.

### 2.7 Western blot

Protein was extracted from mice renal tissues and the concentration was detected by a BCA protein assay kit (Tiangen, Beijing, China). The protein of each group was separated by a 10% SDS-PAGE gel and transferred onto a PVDF membrane. The membrane was blocked with 5% non-fat milk in 1X TBST for 2 h. The membrane was incubated with the primary antibody at 4°C overnight, followed by incubation with the appropriate secondary HRP-IgG antibodies for 1 h. The bands were then detected through chemiluminescence, and their intensities were quantified using densitometry and Image J software.

### 2.8 Real-time PCR

Total RNA was extracted from renal tissue and used for reverse transcription with a Primescript TM RT reagent kit (Takara Biotechnology, Tokyo, Japan). The relative mRNA was quantified in duplicate using SYBR Premix Ex Taq TM II green Master Mix and an ABI Prism 7,300 sequence detection system (Applied Biosystems, United States). The primer sequences used are listed in Supplementary Table S4.

### 2.9 HDAC enzyme activity

To test the enzyme activities of HDAC1 and HDAC2, homogenates of mice renal tissue were analyzed using Mouse HDAC1 Elisa kit (Sino Best Biological Technology, China) and Mouse HDAC2 Elisa kit (Sino Best Biological Technology, China) in accordance with the manufacturer’s instructions.

### 2.10 Reactive oxygen species (ROS) assay of cells

Before detecting the ROS levels, cells grown in the six-well plate were incubated with ROS label (DCFH-DA, 10 mM, Beyotime Biotechnology, China) at 37°C for 20 min in the dark. Then, cells were washed thrice with a serum-free medium and collected in a serum-free medium. The ROS levels of the collected cells were detected and quantified by ACEA NovoCyte flow cytometry (ACEA Biosciences, Agilent, United States) using 488 nm excitation/525 nm emission filters.

### 2.11 TUNEL assessment and PI staining of cells

Terminal deoxynucleotidyl transferase-mediated digoxigenin-deoxyuridine nick-end labeling (TUNEL, Beyotime Biotechnology, China) and Propidium iodide (PI, Beijing solarbio science and technology, China) stains were formulated into working fluids and added to cells that had been treated accordingly. The cells were observed and photographed under a fluorescence microscope. Quantification of apoptotic cells or necrotic cells was performed by taking photos in random fields of wells. ImageJ software was used for quantitative analysis.

### 2.12 Mitochondrial morphology observation by transmission electron microscopy (TEM)

Simply, Cell clusters collected from sesame to mung bean size were removed and placed in a TEM fixative at 4°C. Then the fixed cells were embedded with agarose and fixed at room temperature. The resin blocks were cut into ultrathin sections at 60–80 nm and then, staining was performed. The ultrastructure of the cells was observed by TEM, and the images were collected.

### 2.13 Statistical analysis

The data were presented as mean ± Standard error of mean (SEM). All data were checked for normal distribution firstly, and non-parametric test was performed for the data that do not follow normal distribution. Statistical analysis was carried out using one-way analysis of variance (ANOVA) with GraphPad Prism 8 statistical software. A *p* value less than 0.05 was considered statistically significant.

## 3 Results

### 3.1 Ferroptosis is involved in AKI patients and in cisplatin-induced AKI mice model

**FIGURE 1:**
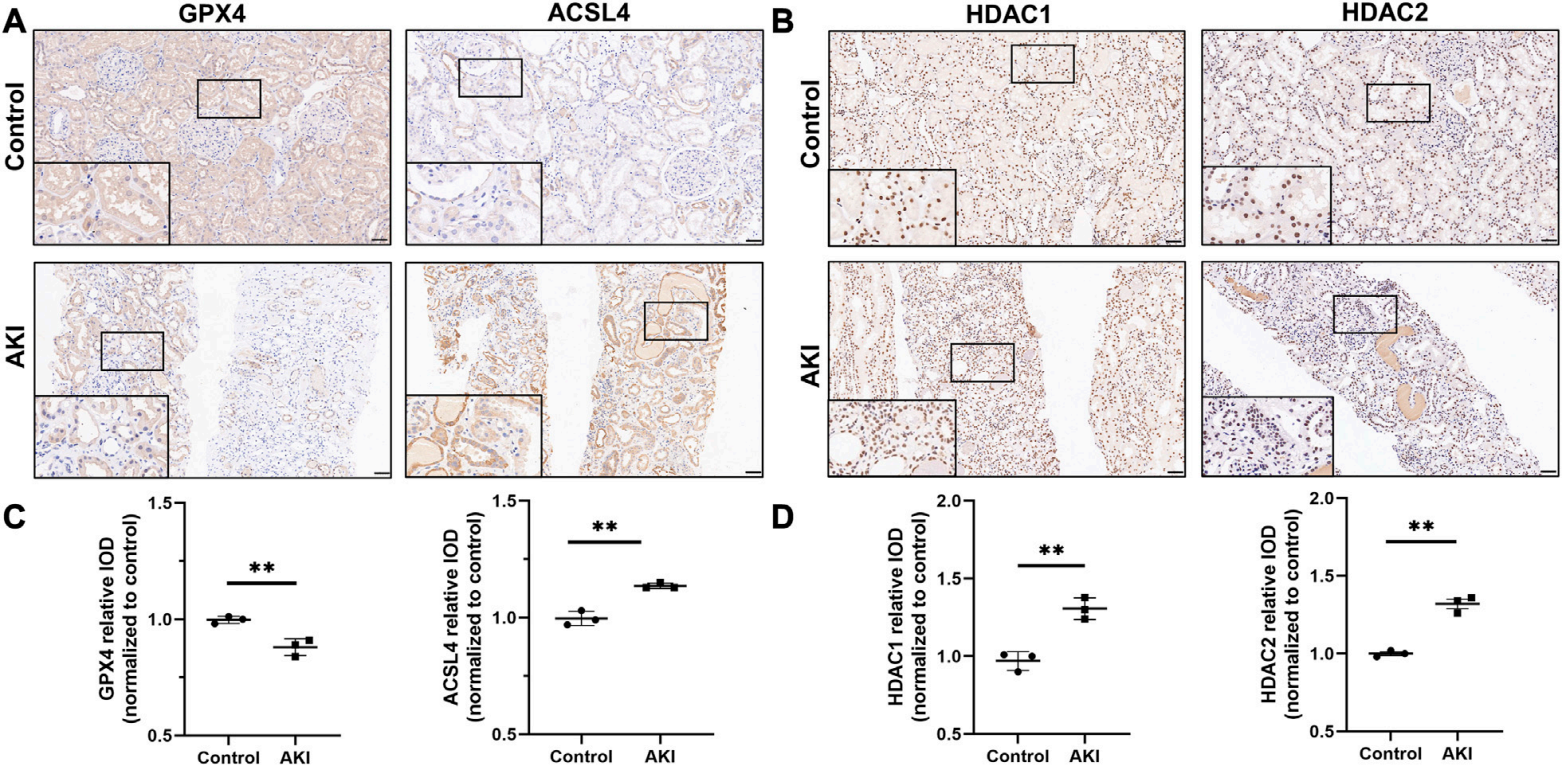
The expression of GPX4, ACSL4, HDAC1 and HDAC2 in acute kidney injury from human biopsies. Representative images of immunohistochemistry of GPX4 and ACSL4 **(A)**, HDAC1 and HDAC2 **(B)** in the healthy control and AKI human kidney biopsy specimens. Scale bar = 50 μm. Quantification of examining positive staining area of GPX4 and ACSL4 **(C)**, HDAC1 and HDAC2 **(D)** in tubulointerstitium normalized to healthy control (Non-tumor kidney tissue from the patients who had renal cell carcinoma and underwent nephrectomy was used as control). n = 3. ✱✱ value *p* < 0.01. Statistically analyzed *via* a one-way ANOVA with Dunnett’s correction.

To investigate the role of ferroptosis in AKI, we selected GPX4 and ACSL4 as markers and analyzed the Nephroseq transcriptomic database (V5). Our findings revealed that GPX4 was downregulated in patients with kidney injury (Supplementary Figure S1A). We also observed a significant negative correlation between *ACSL4* expression in the lupus tubulointerstitium tissue and glomerular filtration rate (GFR) in patients (*R* ^2^ = 0.5610, *p* < 0.0001, Supplementary Figure S1B). Furthermore, a significant positive correlation was found between *ACSL4* expression in tubulointerstitium tissue and serum creatinine level in patients (*R* ^2^ = 0.5377, *p* < 0.0001, Supplementary Figure S1C). We then performed immunohistochemistry of GPX4 and ACSL4 in kidney biopsy tissues from AKI patients. As mentioned before, GPX4 and ACSL4 were expressed in the glomerulus and tubules. Compared with healthy control, GPX4 was significantly decreased and ACSL4 was significantly increased in damaged kidneys (Figure 1A and quantified in 1C).

In order to distinguish the role of ferroptosis in the pathogenesis of cisplatin-induced AKI in mice, we made mice models. In mice after cisplatin-induced, we found an obvious tubular vacuole and cast formation, increased BUN levels, and serum creatinine levels in the cisplatin-induced AKI group (Supplementary Figures S2A–2C). And, Western blot results found upregulated ACSL4, and downregulated GPX4 in the cisplatin-induced group compared to the sham group (Supplementary Figure S2D). These results indicated that ferroptosis may play an essential role in cisplatin-induced AKI.

### 3.2 HDAC plays an important role in AKI and may be related to GPX4 expression

As reported before, the expression of Class Ⅰ HDAC, especially HDAC1 and HDAC2, was significantly increased in AKI patients and mice (Tang et al., 2014; Hyndman, 2020). Due to their highly homologous structures of HDAC1 and 2, they are generally considered to be functionally redundant (Gregoretti et al., 2004). In this study, to explore the role of HDAC1 and HDAC2 in AKI patients, we searched the Nephroseq transcriptomic database (V5). We observed that *HDAC1* and *HDAC2* were upregulated in chronic kidney disease (CKD) kidney tissue (Supplementary Figures S3A, 3B). In addition, we found that the expression of *HDAC1* and *HDAC2* in the lupus tubulointerstitium tissue was significantly negatively correlated with the GFR of patients (*R* ^2^ = 0.8172, *p* = 0.0052, and *R* ^2^ = 0.8651*, p* = 0.0024 respectively, Supplementary Figures S3C, 3E). Furthermore, we observed that the expression of HDAC1 and HDAC2 in the tubulointerstitium tissue was significantly positively correlated with serum creatinine level of patients (*R* ^2^ = 0.3380, *p* = 0.0057 and *R* ^2^ = 0.7877, *p* = 0.0077 respectively, Supplementary Figures S3D, 3F). Then, we performed the immunohistochemistry detection of HDAC1 and HDAC2 in kidney biopsy tissues from AKI patients. As reported earlier, we found that the expression of HDAC1 and HDAC2 in the glomerulus and tubulointerstitium. Compared with healthy control, the expression of HDAC1 and HDAC2 were significantly increased in the renal proximal tubule epithelial cells of damaged kidneys (Figure 1B and quantified in 1D). Thus, in mice after cisplatin, we found HDAC1 and HDAC2 were significantly upregulated in the experiment group compared to the sham group (Supplementary Figure S2D).

VPA can specifically inhibit HDAC1 and HDAC2, and previous studies have demonstrated that VPA treatment improves AKI(Ma et al., 2017), glomerular diseases (Inoue et al., 2019), and UUO(Van Beneden et al., 2013) in mice. To further investigate the relationship between VPA and ferroptosis, we used small interfering RNAs (siRNAs) to silence HDAC1 and HDAC2 in HK-2 cells. We found that decreased GPX4 expression in cells after cisplatin-induced was attenuated in the HDAC silencing group (Supplementary Figure S4). Then, we performed a correlation analysis of HDAC enzyme activity and GPX4 expression in cisplatin-induced mice models. The results showed that HDAC1 and HDAC2 enzyme activity and GPX4 expression are negatively correlated (*R* ^2^ = 0.4777, *p* = 0.0269 and *R* ^2^ = 0.5794, *p* = 0.0105 respectively, Supplementary Figure S5A). As reported before, HDAC plays a role in gene regulation by affecting histone acetylation levels (Zhu et al., 2020). Then we try to further study whether GPX4 can be regulated through epigenetic mechanism. The data of the University of California Santa Cruz (UCSC) genome database showed the enrichment sites for epigenetic marks, and suggested that histone H3 acetylated at lysine 27 (H3K27Ac) may bind to the GPX4 promoter (Supplementary Figure S5B). It is reported that the expression of H3K27Ac increased after the inhibition of HDAC (Caslini et al., 2019). Meanwhile, our study showed that the protein level of H3K27Ac was downregulated in cisplatin-induced injury *in vivo* and *in vitro* by Western blot. These findings suggested that VPA, as an inhibitor of HDAC1 and HDAC2, by regulation of H3K27Ac and GPX4, may play a critical role in the cisplatin-induced AKI. However, the role of VPA in cisplatin-induced AKI mice has not been identified yet.

### 3.3 VPA improves renal injury in cisplatin-induced AKI mice

**FIGURE 2:**
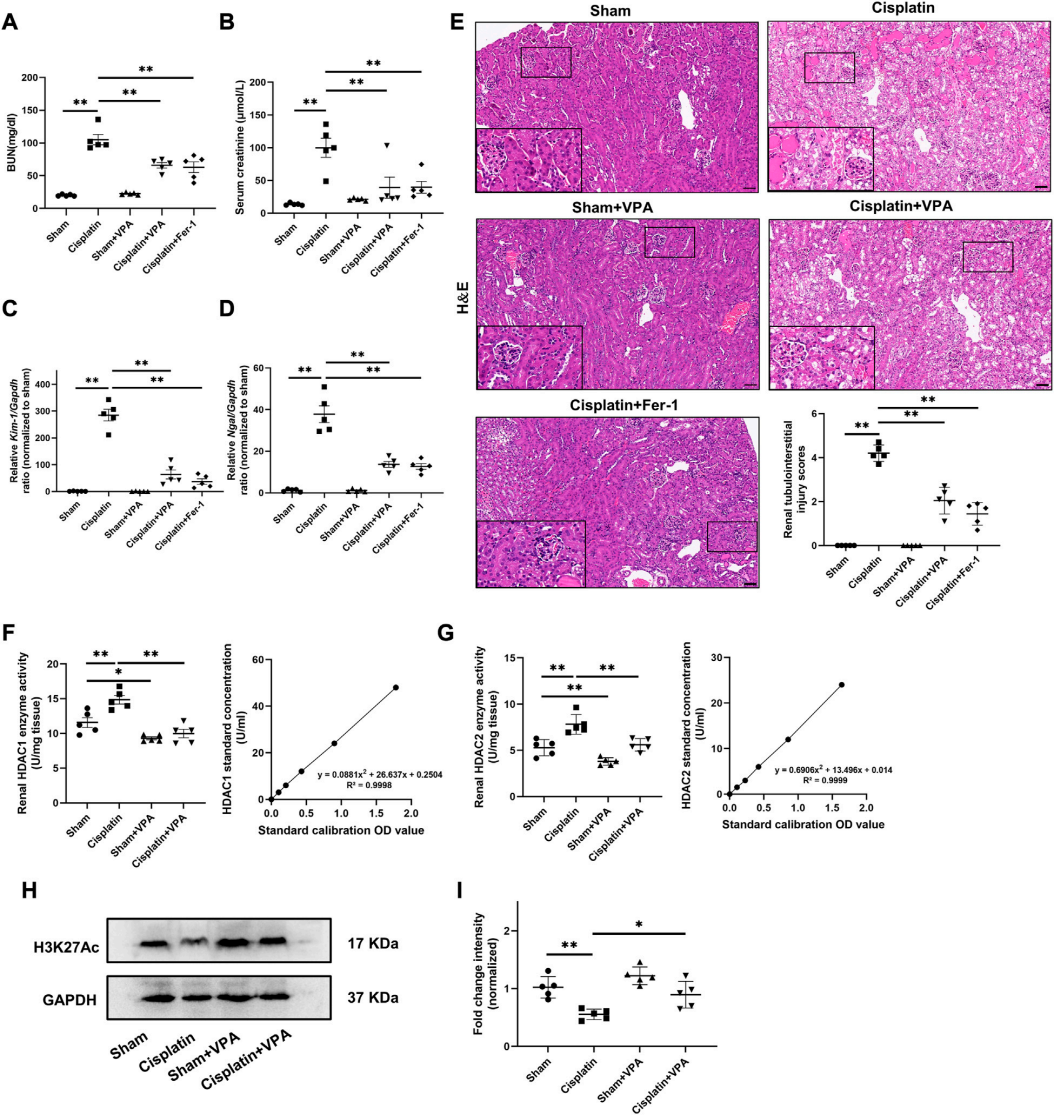
VPA treatment improves renal injury and upregulated the level of H3K27Ac in cisplatin-induced AKI mice. BUN **(A)** and Serum creatinine **(B)** in kidneys of sham, cisplatin, sham + VPA, cisplatin + VPA and cisplatin + Fer-1 group. Quantification of real-time PCR from different groups of mice as indicated, including *Kim-1* **(C)** and *Ngal* **(D)**, respectively. Representative images of H&E staining and quantitative analysis **(E)** of renal tubulointerstitial injury scores in different groups as indicated. Enzyme activities and standard curve line of HDAC1 **(F)** and HDAC2 **(G)** in the renal tissue of mice. Representative images of Western blot **(H)** and quantitative analysis **(I)** of the level of H3K27Ac of kidneys in different groups as indicated. n = 5. Scale bar = 50 μm ✱✱ value *p* < 0.01, ✱ value *p* < 0.05. Statistically analyzed *via* a one-way ANOVA.

To further explore the contribution of VPA in the pathogenesis of cisplatin-induced AKI mice leading to ferroptosis, the inhibitor of HDAC (VPA) or the inhibitor of ferroptosis (Fer-1) was used to carry out the following experiments. To investigate the effect of VPA or Fer-1 in mice after cisplatin, mice were intraperitoneally injected with 200 mg/kg of VPA or 1 mg/kg of Fer-1, depending on their body weight after randomization. When calculating the ratio of left and right kidney weight to body weight and body weight of mice, the cisplatin group had a larger volume and a lower body weight of kidney than that of the sham group but the volume decreased and weight increased after Fer-1 treatment, while this was not found after VPA treatment (Supplementary Figure S6). As is shown in Figures 2A, B, the BUN and serum creatinine were increased in the cisplatin group, which were significantly improved after treatment with VPA or Fer-1. Then, the mRNA expression of kidney injury molecule 1 (KIM-1) and neutrophil gelatinase-associated lipocalin (NGAL) in real-time PCR was decreased after VPA or Fer-1 treatment (Figures 2C, D). In accordance with this, we found that in the cisplatin-induced mice, significant areas of tubular damage (extensive tubular cell death, severe cell shedding, interstitial edema, cast formation, and brush border destruction) were mitigated by VPA or Fer-1, as shown by H&E staining of renal tissues. Also, we performed the pathological analysis of renal tubular injury, and found that VPA or Fer-1 therapy can improve cisplatin-induced AKI (Figure 2E).

Also, since the role of VPA is closely related to HDAC enzyme activity, we examined the enzyme activities of HDAC 1 and 2. Results showed that HDAC 1 and 2 activities in the renal tissue of the cisplatin group increased significantly compared to that in the sham group, which decreased after VPA treatment (Figures 2F, G). Then, we examined the level of H3K27Ac in the cisplatin-induced AKI model through Western blot. Compared with the sham group, the expression of H3K27Ac was significantly downregulated in the cisplatin group, which was increased after VPA treatment (Figures 2H, I).

### 3.4 VPA attenuates ferroptosis and protected cisplatin-induced AKI mice

**FIGURE 3:**
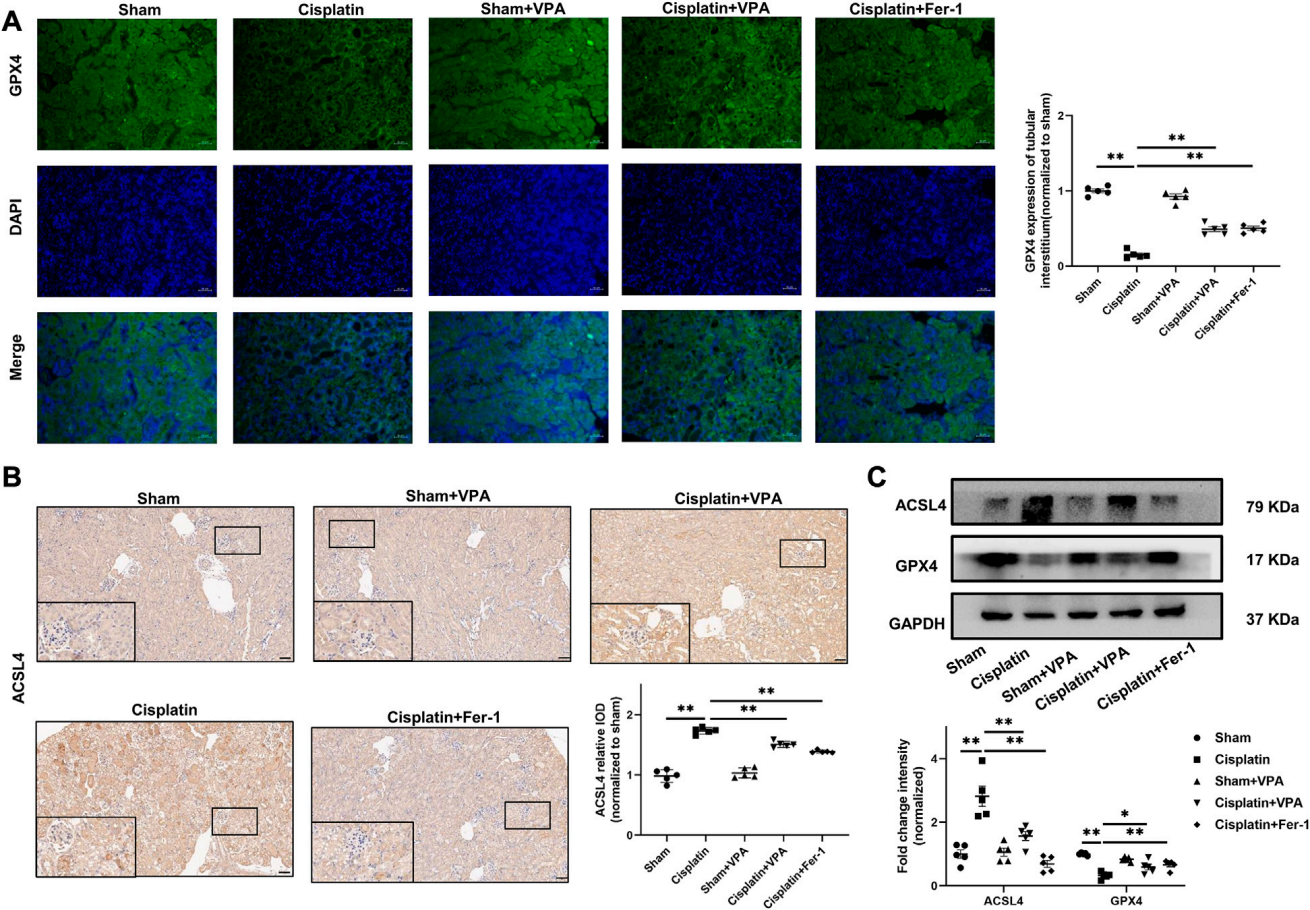
VPA treatment attenuates expression of ferroptosis-related proteins in cisplatin-induced AKI mice. Representative images of immunofluorescence of GPX4 in the tubulointerstitium of sham, cisplatin and VPA or Fer-1 treatment groups. Quantification of examining positive staining area of GPX4 in the tubulointerstitium normalized to sham group **(A)**. Immunohistochemical staining and semi-quantitative analysis for expression of ACSL4 **(B)** in different groups as indicated. Representative images of Western blot from different groups as indicated, including ACSL4 and GPX4, respectively **(C)**. Scale bar = 50 μm. n = 5. ✱✱ value *p* < 0.01, ✱ value *p* < 0.05. Statistically analyzed *via* a one-way ANOVA.

Recent studies show ferroptosis is involved in cisplatin-induced AKI(Hu et al., 2020). In order to further prove that VPA play an important role in cisplatin-induced AKI by inhibiting ferroptosis, we examined the expression of two important enzymes, GPX4 and ACSL4, which regulate ferroptosis by IF, IHC, and Western blot. As is shown in Figures 3A, C, renal GPX4 expression decreased after cisplatin injury, whereas VPA significantly improved GPX4 expression. The effect was similar to the Fer-1 treatment. Then, IHC and Western blot analysis showed that the expression of ACSL4 in mice was significantly increased after cisplatin treatment, which was inhibited by VPA or Fer-1 treatment (Figures 3B, C).

**FIGURE 4:**
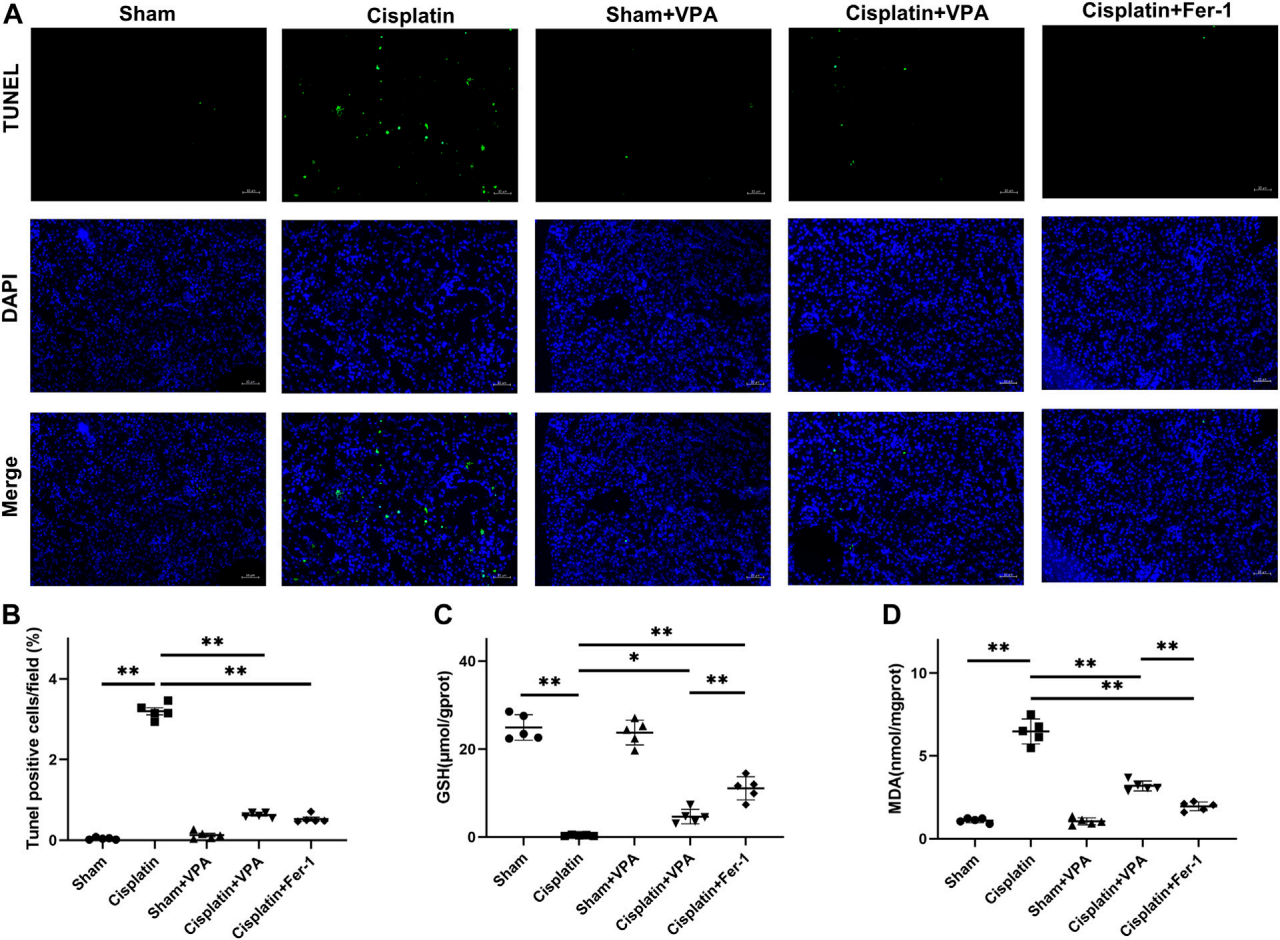
VPA treatment improves oxidative stress in cisplatin-induced AKI mice. **(A)** TUNEL fluorescence staining and statistical analysis of cell death rates in each group at 72 h after cisplatin treatment. Green fluorescence in pictures represent positive signals of apoptosis cells, while blue fluorescence represents nuclear staining. Quantitative analysis of TUNEL fluorescence staining in different groups **(B)**. The production of GSH **(C)** and MDA **(D)** in cisplatin-induced AKI mice. Scale bar = 10 μm. n = 5. ✱✱ value *p* < 0.01, ✱ value *p* < 0.05. Statistically analyzed *via* a one-way ANOVA.

In addition, apoptotic cells and lipid peroxidation levels were determined. We found VPA could remarkably reduce apoptosis induced by cisplatin measured by TUNEL stain (Figures 4A, B). Compared with the sham group, the GSH levels decreased significantly in the cisplatin group and improved after VPA or Fer-1 treatment (Figure 4C). MDA, a marker of oxidative stress, was enhanced in the cisplatin group but suppressed by VPA or Fer-1 treatment (Figure 4D). Furthermore, the efficacy of VPA treatment in GSH and MDA were significant, but it was weaker than the Fer-1 treatment in cisplatin-induced AKI mice. These data suggest that the protective effect of VPA is achieved by inhibiting ferroptosis, and VPA could be a valuable ferroptosis inhibitor for AKI.

### 3.5 VPA alleviated cisplatin-induced ferroptosis in HK-2 cells

**FIGURE 5:**
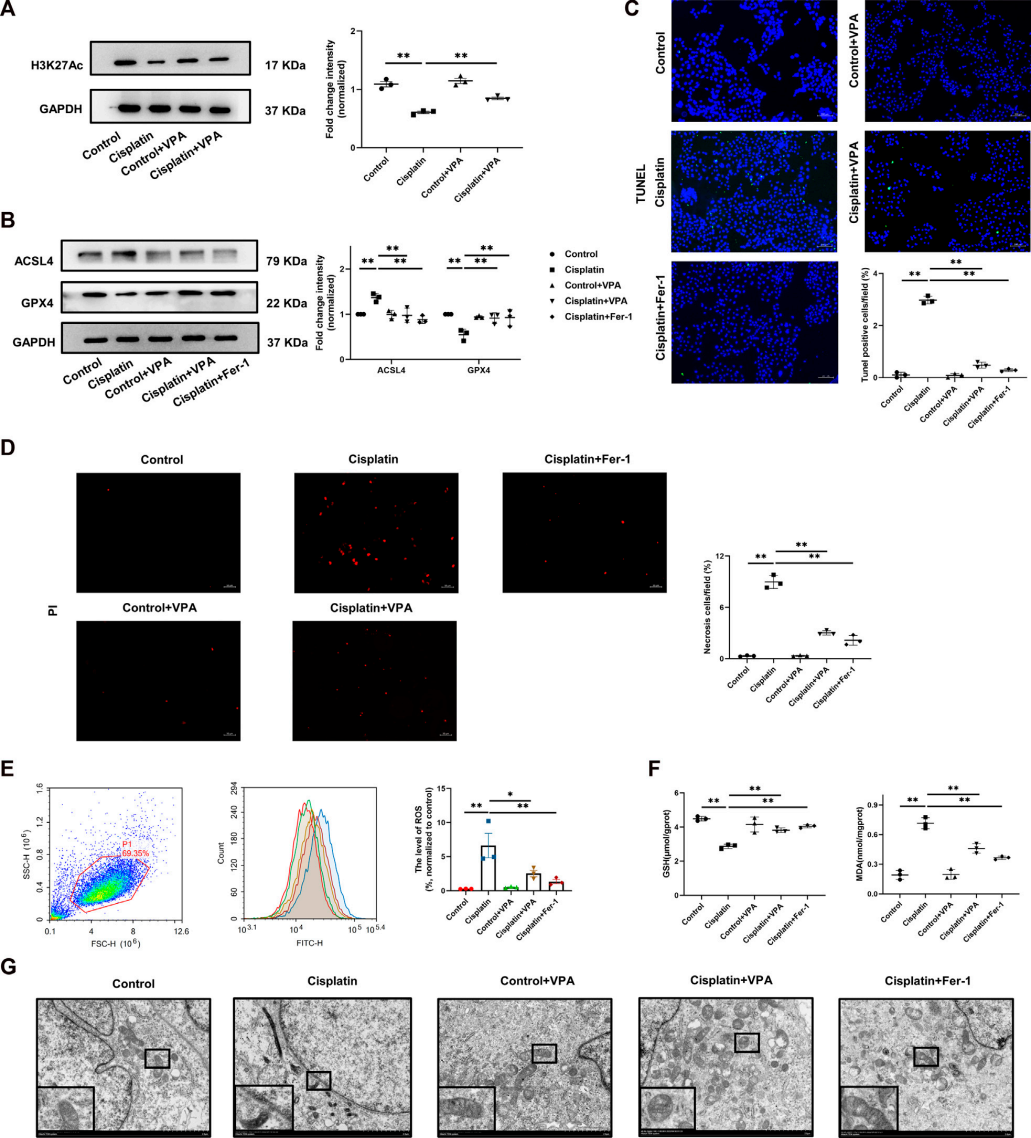
The cisplatin-induced ferroptosis is improved by VPA in HK-2 cells. Representative images of Western Blot and quantification of H3K27Ac in HK-2 cells stimulated by cisplatin **(A)**. Representative images of Western blot and quantification of ACSL4 and GPX4 in HK-2 cells stimulated by cisplatin **(B)**. PI staining (red) and statistical analysis of cell death in each group in HK-2 cells stimulated by cisplatin. Scale bar = 50 μm **(C)**. TUNEL fluorescence staining and statistical analysis of apoptosis cells in each group in HK-2 cells stimulated by cisplatin. Green fluorescence in pictures represent positive signals of apoptosis cells, while blue fluorescence represents nuclear staining **(D)**, Scale bar = 200 μm. The production of ROS was detected by ROS assay kit and was present as FITC-H value in P1 region **(E)**. The production of GSH and MDA in cisplatin-stimulated HK-2 cells **(F)**. Transmission electron microscopy of HK-2 cell treated with cisplatin, VPA or Fer-1 **(G)**, Scale bar = 2 μm. n = 3. ✱✱ value *p* < 0.01, ✱ value *p* < 0.05. Statistically analyzed *via* a one-way ANOVA.

To better understand the role of VPA and ferroptosis in kidney cells, we performed VPA treatment in cisplatin-induced HK-2 cells. First, we observed a significant increase of HDAC1 and HDAC2 in HK-2 cells after stimulation with 10 μM cisplatin for 24 h (Supplementary Figure S7). The level of H3K27Ac was decreased after cisplatin and improved in HK-2 cells after VPA (1 mM) treatment (Figure 5A). Meanwhile, pretreatment of HK-2 cells with VPA markedly mitigated the expression of GPX4 and ACSL4 in HK-2 cells induced by cisplatin (Figure 5B). Through PI and TUNEL staining, we found that VPA and Fer-1 significantly reduced cisplatin-induced cell death including apoptotic cells and necrotic cells (Figures 5C, D). Then, the expression of ROS in HK-2 cells was represented by the mean fluorescence intensity (FITC-A) in the region of P1. As is shown in Figure 5E, ROS levels in the cisplatin group were higher compared to the control group, but suppressed by VPA or Fer-1 treatment. Also, compared with the control group, the GSH levels decreased significantly in the cisplatin group and improved after VPA or Fer-1 treatment. MDA levels were enhanced in the cisplatin group but suppressed by VPA or Fer-1 treatment (Figure 5F). Moreover, electron microscopy observation showed that compared with the control group, cisplatin-induced HK-2 cells exhibited more swollen mitochondria, outer mitochondrial membrane rupture, and partial cristae breakdown. These changes were also found to be significantly mitigated by VPA or Fer-1 treatment (Figure 5G).

### 3.6 The protective effect of VPA is weakened by GPX4 deficiency

**FIGURE 6:**
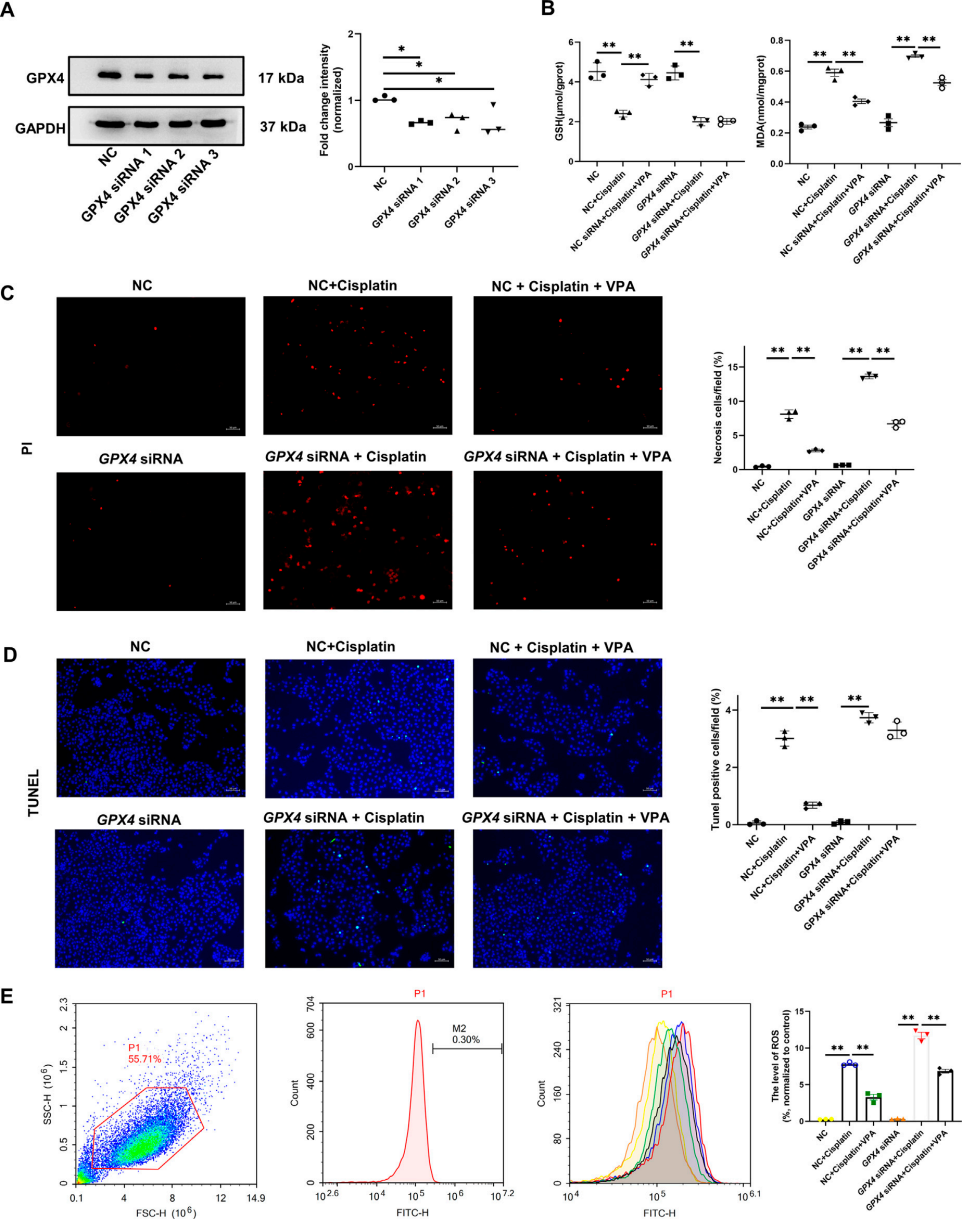
The protective effect of VPA is weakened by GPX4 deficiency. Representative images of Western blot and quantification of the level of GPX4 in HK-2 cells **(A)**. The production of GSH and MDA in cisplatin-stimulated HK-2 cells with GPX4 siRNA **(B)**. PI staining (red) and statistical analysis of cell death in each group in HK-2 cells. Scale bar = 50 μm **(C)**. TUNEL fluorescence staining and statistical analysis of apoptosis cells in each group in HK-2 cells. Green fluorescence in pictures represent positive signals of apoptosis cells, while blue fluorescence represents nuclear staining, Scale bar = 50 μm **(D)**. The production of ROS was detected by ROS assay kit and was present as FITC-H value in P1 region **(E)**. ✱✱ value *p* < 0.01, ✱ value *p* < 0.05. Statistically analyzed *via* a one-way ANOVA.

In order to further demonstrate whether the ferroptosis-inhibiting effect of VPA depends on the regulation of GPX4, we conducted an *in vitro* experiment on HK-2 cells. We transfected three *GPX4* siRNAs and negative control siRNA in HK-2 cells. The respective silencing efficiency of *GPX4* siRNAs was shown in Figure 6A. Then we selected the siRNA with the highest inhibition efficiency for the following experiments. As is shown in Figures 6B–E, cisplatin significantly induced less GSH and more MDA production, more apoptotic cells and necrotic cells, and ROS accumulation, which were ameliorated by pretreatment with VPA, consistent with the previous results. However, this effect was largely eliminated after inhibiting the expression of GPX4 by siRNA. These data showed us that the protection of VPA may depend on GPX4.

## 4 Discussion

In this study, we confirmed that ferroptosis plays an important role in cisplatin-induced AKI in human and mice, after observing of expression of antiferoptotic gene (GPX4), proferoptotic gene (ACSL4), and lipid peroxidation *in vivo* and vitro. Moreover, we first identified that VPA, which improved tubular injury in renal and inhibited the accumulation of lipid peroxidation in the cisplatin-induced model, exerts renoprotection by inhibiting ferroptosis *via* regulation of GPX4. Therefore, we found that VPA could be a potential ferroptosis inhibitor.

As mentioned earlier, cisplatin is well-known as an effective chemotherapeutic agent, but usually, damages the kidneys and lacking available treatments (Zhang et al., 2021). Over the years, it is reported that the mechanisms of cisplatin-induced AKI in dysregulation of oxidative stress (Volarevic et al., 2019), mitochondrial dysfunction (Guo et al., 2018), endoplasmic reticulum stress (Ye et al., 2015), inflammatory responses (Wang et al., 2018), apoptosis (Sen et al., 2017), necrosis (Xu et al., 2015), and autophagy (Kaushal and Shah, 2016) have been extensively studied. Before ferroptosis was defined, a study from Baliga R (Baliga et al., 1998) indicated that iron plays a critical role in mediating kidney injury *via* hydroxyl radical in cisplatin-induced AKI. Ferroptosis, a novel mode of cell death associated with mitochondrial dysregulation, is closely related to oxidative stress and inflammation (Dixon et al., 2012). Therefore, ferroptosis might be important in cisplatin-induced AKI.

In recent years, only a few studies have found the contribution of ferroptosis in cisplatin-induced AKI, while verifying the ameliorating effects of different molecules on ferroptosis. The process of ferroptosis is featured by abnormal iron metabolism and the accumulation of ROS and lipid peroxidation products, which can be inhibited by iron chelators and lipid peroxidation inhibitors, such as ferrostatin and zileuton (Xie et al., 2016). A study reported by Yashpal S. Kanwar et al. (Deng et al., 2019) indicated that Fer-1 treatment improves tubular damage in cisplatin-induced AKI, and alterations in GPX4 expression, NADPH, and GSH levels were observed in cisplatin-treated HK-2 cells, while regulated by Myo-inositol oxygenase. Another study from Wei Zhang et al. (Hu et al., 2020) showed that vitamin D receptor activation protects against cisplatin-induced AKI by inhibiting ferroptosis through regulation of GPX4. Consistent with their results, our study also found that ferroptosis inhibition might be an effective treatment for cisplatin-induced AKI which *via* regulation of GPX4. Overall, finding new targets or drugs for clinical treatment to improve ferroptosis in cisplatin-induced AKI patients still need further research.

VPA (inhibitor of HDAC1 and HDAC2), which was used in the clinic for many years, has been found to play a protective effect on a variety of kidney diseases. Most of the study reported focus on chronic kidney diseases such as IRI (Zheng et al., 2014), LPS(Shang et al., 2010), UUO model (Kawaoka et al., 2017), and diabetic nephropathy (Khan et al., 2015; Sun et al., 2016). However, only a few articles have investigated the protective effect of VPA in cisplatin-induced AKI, and none of the studies confirmed whether VPA improves cisplatin-induced AKI by inhibiting ferroptosis. Currently, a previous study observed that VPA can ameliorate cisplatin-induced AKI by suppressing BMP-7 and promoting apoptosis in mice (Ma et al., 2017). Another study found that VPA by upregulated H3K27Ac orchestrates IL-9 mediated renoprotection in cisplatin-induced AKI(Jiang et al., 2020). Studies mentioned before showed that VPA treatment improve kidney injury, promoted tubular proliferation, and decreased cell death and inflammation after cisplatin (Ma et al., 2017; Jiang et al., 2020; Zhu et al., 2020), which was consistent with our data. In this study, we firstly identified that VPA suppressed the expression of ACSL4, lipid peroxidation, and mitochondria damage, while reversing the expression of GPX4 in cisplatin-induced AKI. Clearly, the protective effect of Fer-1 was evident in animal studies, but its translation into clinical therapy remains to be explored. As an FDA-approved drug, VPA is effective in a variety of animal models and can also significantly delay the decline of GFR in patients in cohort studies (Inoue et al., 2019). Therefore, AKI patients, especially those with epilepsy, may benefit more from VPA treatment.

Then, the mechanism of VPA on ferroptosis in cisplatin-induced AKI needs further study. HDAC1 and HDAC2, as deacetylases of H3K27Ac are involved in various cellular processes such as apoptosis and DNA replication by regulating acetylation (Wang et al., 2022). As reported in these researches (Jiang et al., 2020; Zhu et al., 2020), affecting H3K27Ac attenuate kidney injury after cisplatin. A study from Punithavathi Ranganathan (Ranganathan et al., 2016) found that HDAC 1 and HDAC2 increased significantly after cisplatin and HDAC inhibitor, like trichostatin (TSA), MS-275, FK-228, and Tubastatin A, suppressed cisplatin-induced kidney dysfunction by increasing levels of acetylated protein of histone H3/H4. Consisting with our study, we observed that VPA might achieve renal protection by improving the expression of H3K27Ac in mice and cell models. Meanwhile, after inhibiting GPX4 by siRNA *in vitro*, we speculated that VPA acts its protective effect by regulating GPX4. As predicted by the UCSC database, VPA may act as an antiferoptotic agent by affecting the binding of H3K27Ac to GPX4. However, more experiments are needed to confirm this. Also, this study lacked changes in mitochondria in kidney tissue under electron microscopy. And there are no relevant indicators of iron metabolism in our study, which needs to be further.

All things considered, our study’s findings confirm the crucial role of ferroptosis in cisplatin-induced AKI, and indicate that VPA can mitigate cisplatin-induced AKI and suppress ferroptosis by regulating GPX4. These results suggest that ferroptosis is a promising target for preventing and treating cisplatin-induced AKI, and that VPA may be a possible therapy, particularly for epilepsy patients with cisplatin-induced AKI. However, further research is still needed to fully explore this potential treatment option.

## Data Availability

The original contributions presented in the study are included in the article/Supplementary Materials, further inquiries can be directed to the corresponding authors.
