# Supplementary material for: VPA improves ferroptosis in tubular epithelial cells after cisplatin-induced acute kidney injury
Source: Front Pharmacol. 2023 Apr 19;14:1147772. doi: 10.3389/fphar.2023.1147772 (PMC10155836; doi:10.3389/fphar.2023.1147772)
Supplement: Supplementary file 1 [file DataSheet2.pdf]

Supplementary Table 1. Reagents used in this study.

Reagents	Supplier	Catalog number
cis-Diammineplatinum dichloride	Aladdin, China	D109812
Valproic acid	MedChemExpress, USA	HY-10585A
Ferrostain-1	MedChemExpress, USA	HY-100579
Puromycin dihydrochloride hydrate	Beijing solarbio science and technology, China	PB230
Blood urea nitrogen assay kit	Nanjing Jiancheng Bioengineering Institute, China	C013-2-1
Creatinine assay kit	Nanjing Jiancheng Bioengineering Institute, China	C011-2-1
Hematoxylin and eosin staining kit	Servicebio, Wuhan, China	GP1032
Mouse HDAC1 Elisa kit	Sino Best Biological Technology Shanghai, China	YX-E10432
Mouse HDAC2 Elisa kit	Sino Best Biological Technology Shanghai, China	YX-E10441
Reduced glutathione (GSH) assay kit	Nanjing Jiancheng Bioengineering Institute, China	A006-2-1
Malondialdehyde (MDA) assay kit	Nanjing Jiancheng Bioengineering Institute, China	A003-1-2
Cell malondialdehyde (MDA) assay kit	Nanjing Jiancheng Bioengineering Institute, China	A003-4-1
Reactive oxygen species (ROS) assay kit	Beyotime biotechnology, China	S0033M
One step TUNEL apoptosis assay kit	Beyotime biotechnology, China	C1088
Propidium iodide (PI)	Beijing solarbio science and technology, China	CA1630

Supplementary Table 2. Antibodies used in this study.

Antibodies	Dilution and supplier	Catalog number	Application
HDAC1	1:1000/1:200, Proteintech, Wuhan, China	66085-1-Ig	WB/IHC
HDAC2	1:1000/1:200, Abcam, UK	ab32117	WB/IHC
ACSL4	1:1000/1:200, Abcam, UK	ab155282	WB/IHC
GPX4	1:1000/1:200, Abcam, UK	ab125066	WB/IF
Histone H3 (acetyl K27)	1:1000, Abcam, UK	ab4729	WB
GAPDH	1:5000, Proteintech, Wuhan, China	60004-1-Ig	WB
Goat anti-Rabbit IgG	1:5000/1:200, Beijing TDY Biotech, China	S004F	WB/IHC
Goat anti-Mouse IgG	1:5000/1:200, Beijing TDY Biotech, China	S001F	WB/IHC

Supplementary Table 3. Sequences of the three small interfering RNA targeting human *GPX4* mRNA.

Species	Gene	Sequence (5'-3')
Human	*GPX4*<br>SiRNA-homo-273	sense 5'-GACCGAAGUAAACUACACUTT
		antisense 5'-AGUGUAGUUUACUUCGGUCTT
	GPX4<br>SiRNA-homo-380	sense 5'-GGAGUAACGAAGAGAUCAATT
		antisense 5'-UUGAUCUCUUCGUUACUCCTT
	GPX4<br>SiRNA-homo-479	sense 5'-GGAAGUGGAUGAAGAUCCATT
		antisense 5'-UGGAUCUUCAUCCACUUCCTT

Supplementary Table 4. Primers for Real-time PCR

Species	Gene	Sequence (5’-3’)
Mouse	Gapdh	sense 5’-TGACCTCAACTACATGGTCTACA-3’
		antisense 5’-CTTCCCATTCTCGGCCTTG-3’
	Kim-1	sense 5’-ACATATCGTGGAATCACAACGAC
		antisense 5’-ACTGCTCTTCTGATAGGTGACA
	Ngal	sense 5’-GCCCAGGACTCAACTCAGAA
		antisense 5’-GACCAGGATGGAGGTGACAT
