# Supplementary material for: VPA improves ferroptosis in tubular epithelial cells after cisplatin-induced acute kidney injury
Source: Front Pharmacol. 2023 Apr 19;14:1147772. doi: 10.3389/fphar.2023.1147772 (PMC10155836; doi:10.3389/fphar.2023.1147772)

Supplementary Figure

Supplementary figure 1: The decreased expression of GPX4 in renal dysfunction patients and the correlation between *GPX4* or *ACSL4* expression and GFR or serum creatinine in tubulointerstitial injury from Nephroseq transcriptomic database (V5). ∗∗ value *P* < 0.01.

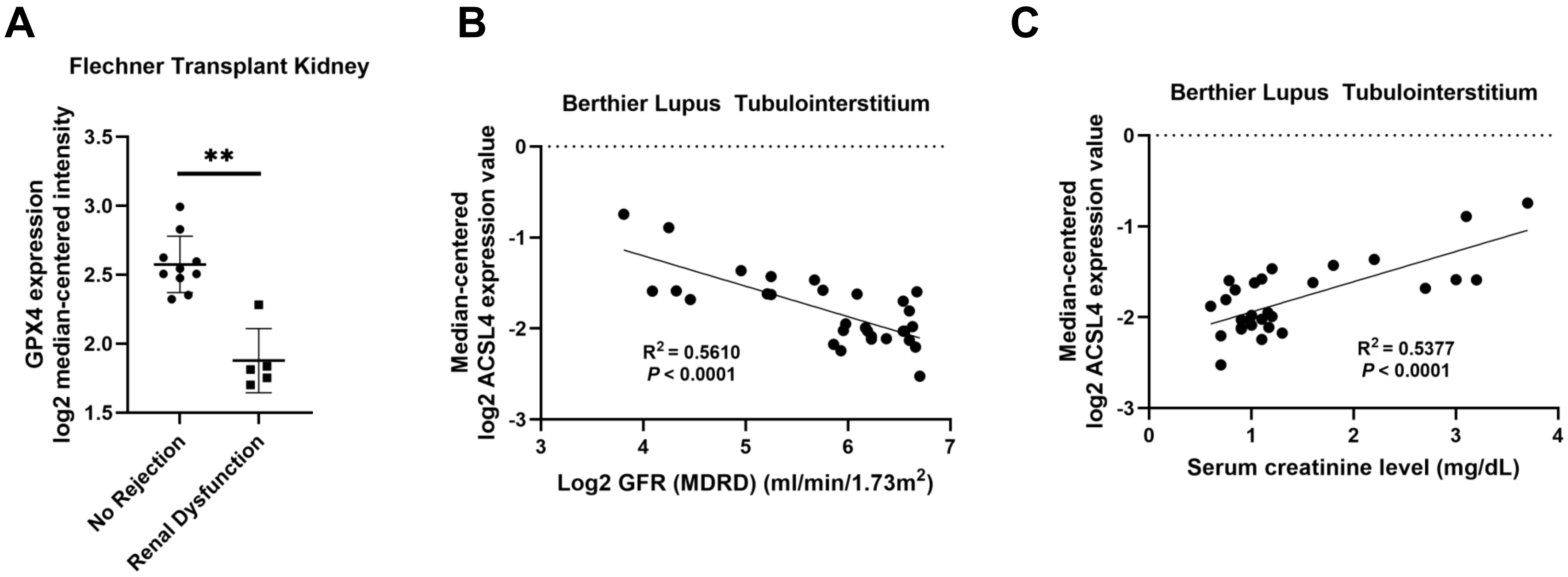

Supplementary figure 2: Changed expression of HDAC1, HDAC2, GPX4 and ACSL4 in the kidney of Cisplatin-induced mice. Representative images of H&E staining and quantitative analysis of renal tubulointerstitial injury scores in kidneys of sham and Cisplatin group (A and B). BUN and Serum creatinine (C) in different groups as indicated. Representative images of Western Blot and quantitative analysis of the expression of HDAC1, HDAC2, GPX4 and ACSL4 of kidneys in two groups (D). Scale bar = 50 μm. n=3. ∗∗ value *P* < 0.01, ∗ value *P* < 0.05. Statistically analyzed via a one-way ANOVA with Dunnett's correction.

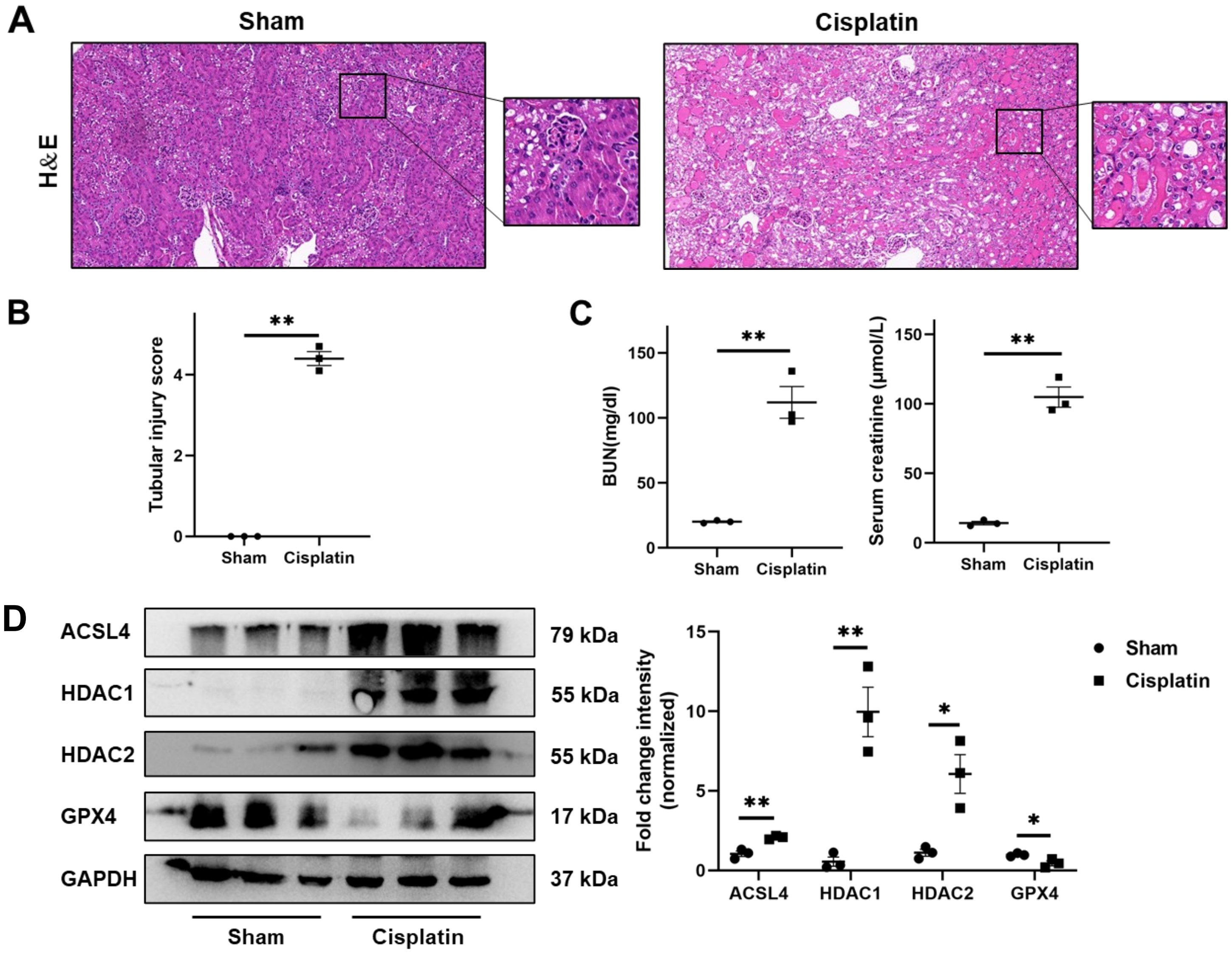

Supplementary figure 3: Increased expression of HDAC1 and HDAC2 in CKD patients and the correlation between *HDAC1* or *HDAC2* expression and GFR or serum creatinine in tubulointerstitial injury from Nephroseq transcriptomic database (V5). ∗∗ value $P < 0.01$.

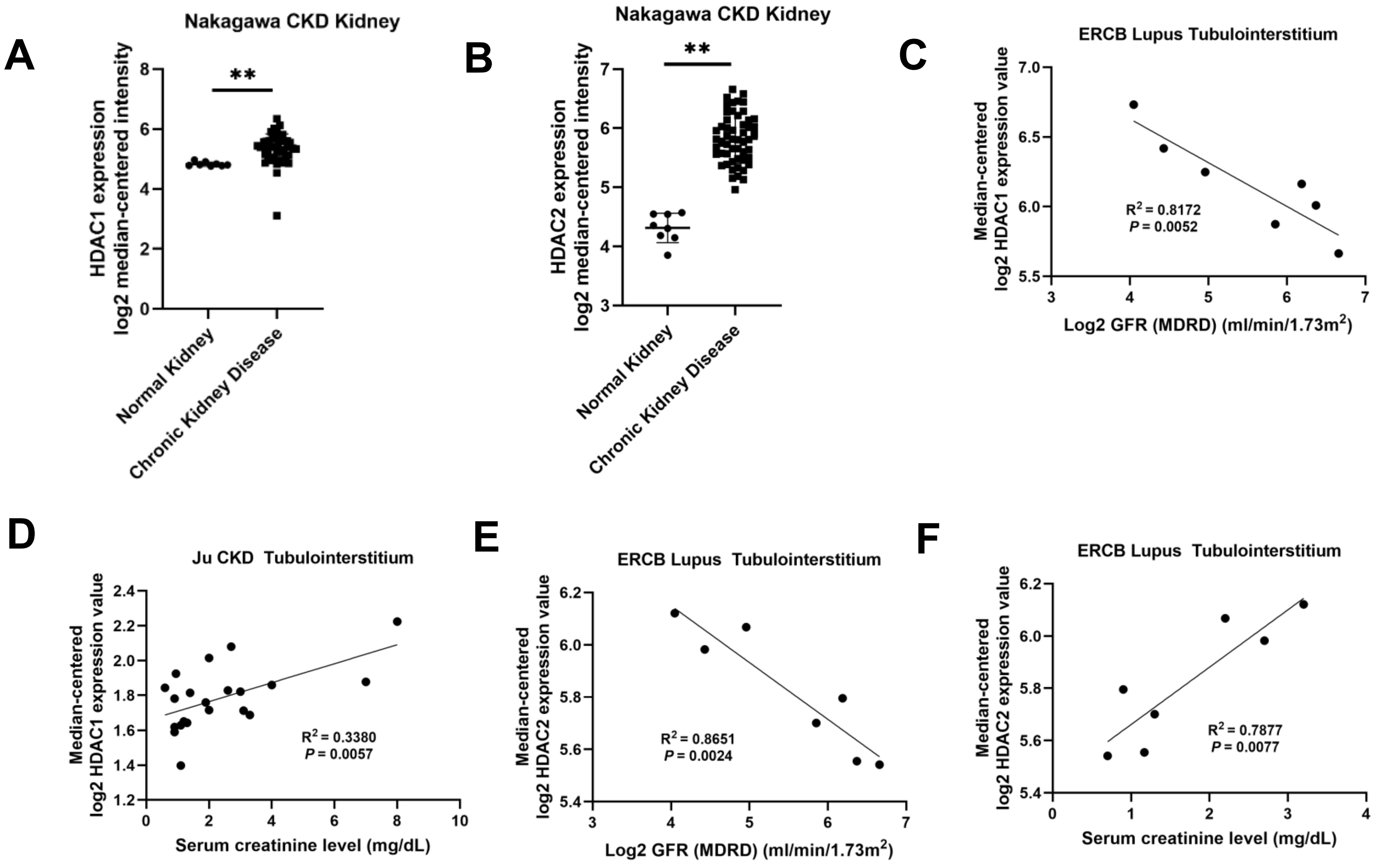

Supplementary figure 4: Changed expression of GPX4 in HK-2 cells induced by cisplatin with HDAC siRNAs. Representative images of western blot and quantification of the level of HDAC1(A) and HDAC2 (B) in HK-2 cells with HDAC siRNAs. Representative images of western blot and quantification of the level of GPX4 in cisplatin-stimulated HK-2 cells with HDAC1 (C) and HDAC2 (D) siRNA. n=3. ✱✱ value $P < 0.01$, ✱ value $P < 0.05$. Statistically analyzed via a one-way ANOVA with Dunnett's correction.

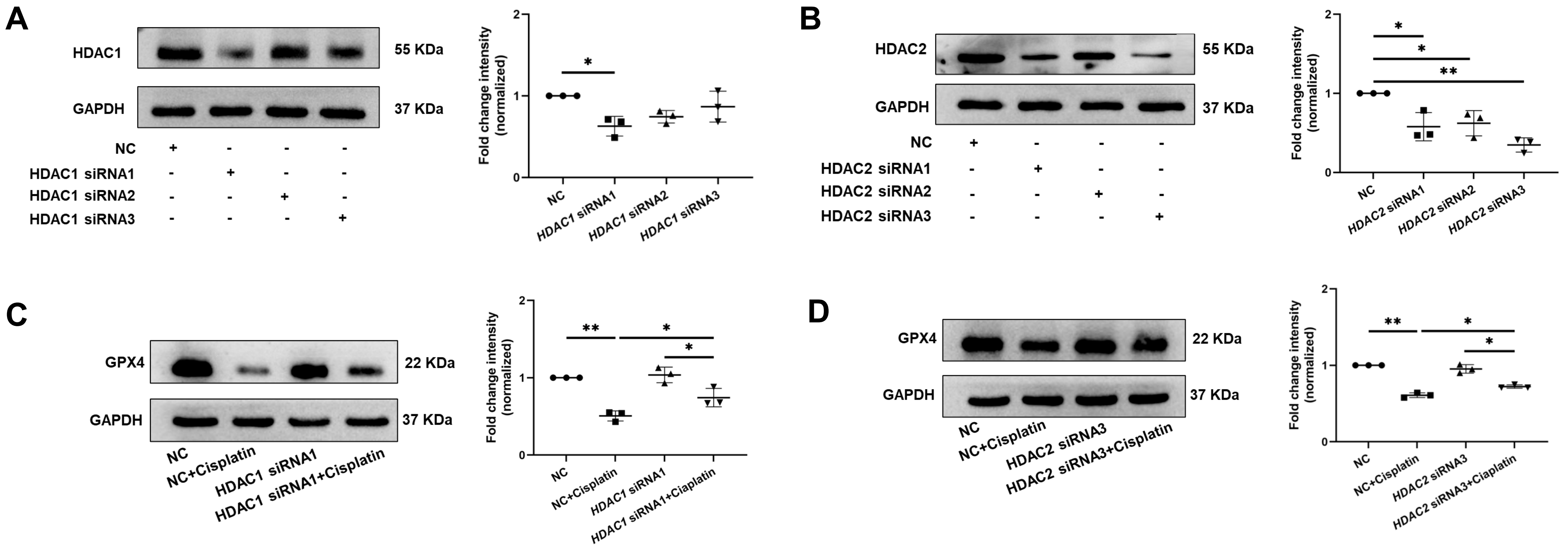

Supplementary figure 5: HDAC enzyme activity and GPX4 expression may be related. (A) HDAC enzyme activity is negatively correlated with GPX4 expression in mice after cisplatin (n = 10). (B) Predicted acetylated H3K27 binding site to GPX4 from UCSC database (GRCh38/hg38).

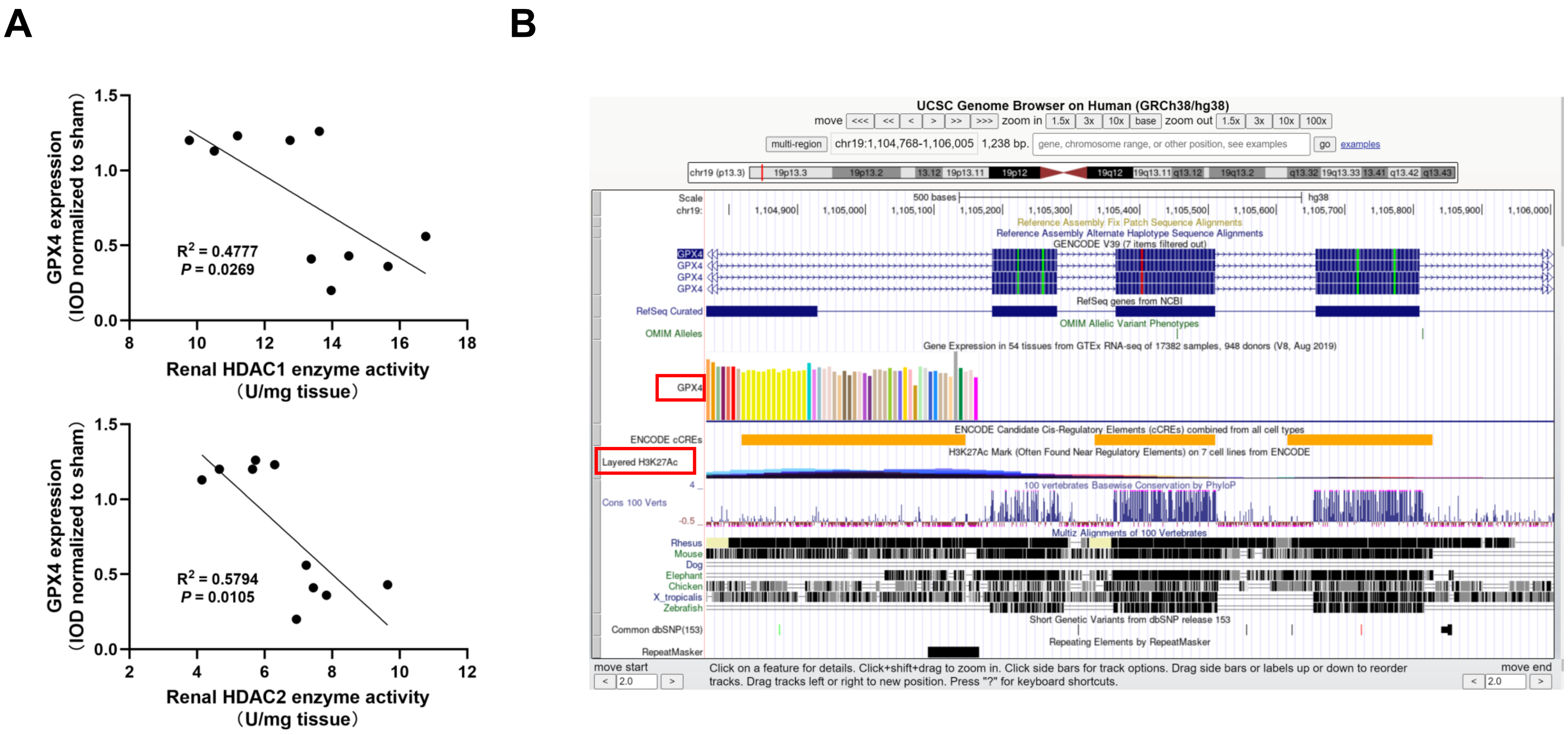

Supplementary figure 6: Change in renal index and weight of cisplatin-induced AKI mice after VPA treatment. Body weight of mice was measured after randomization and before sampling (A). Renal index values the ratio of left (B) and right (C) kidney weight to body weight of mice. n=5. ∗∗ value *P* < 0.01, ∗ value *P* < 0.05. Statistically analyzed via a one-way ANOVA with Dunnett‘s correction.

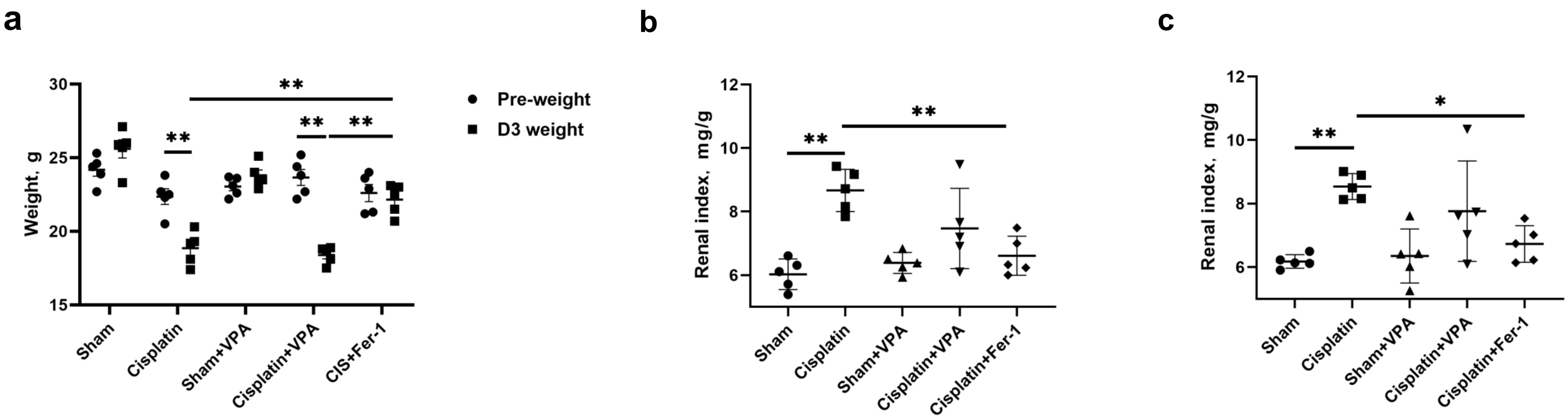

Supplementary figure 7: Change expression of HDAC in HK-2 cells treatment by cisplatin. Representative images of Western Blot (A) and quantitative analysis (B) of the expression of HDAC1 and HDAC2 of HK-2 cells in two groups.

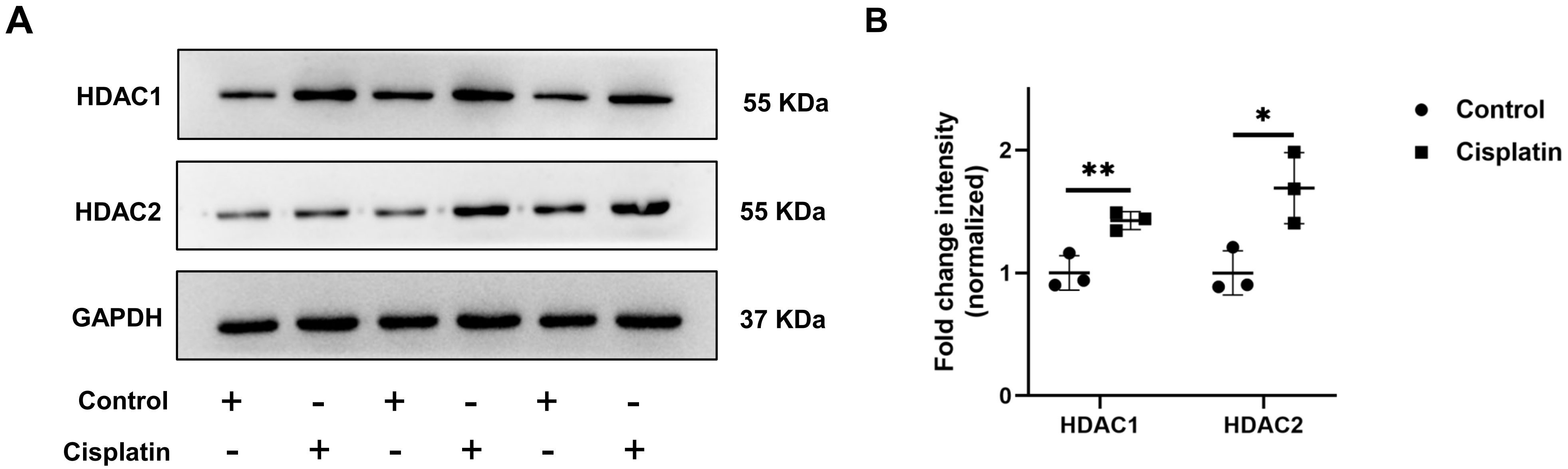

Supplement: Supplementary file 2 [file DataSheet1.pdf]
